# Recurrent ischial pressure ulcer resolved with a novel tissue adhesive: a case report

**DOI:** 10.1186/1752-1947-9-20

**Published:** 2015-02-20

**Authors:** Ingo Kuhfuss, Alessandro Cordi, Philip Zeplin

**Affiliations:** 7 Klinik für Plastische und Ästhetische Chirurgie, Handchirurgie St Josefs-Hospital, Dreieckstr. 17, 58097 Hagen, Germany; 8 Universitätsklinikum Leipzig; Klinik für Orthopädie, Unfall- und Plastische Chirurgie, Liebigstr. 20, 04103 Leipzig, Germany

**Keywords:** Pressure sore, Flap surgery, Surgical adhesive

## Abstract

**Introduction:**

Patients with stage III and IV pressure ulcers requiring surgical reconstruction remain a challenge. Extended hospitalization, and high costs of care per patient episode due to high rates of complications and recurrence, make efforts to reduce these rates of utmost importance to the medical community in general. We report a case in which two prior attempts at surgical resolution had failed, and which was successfully resolved with the aid of a new tissue adhesive designed for the closure of dead space. To the best of our knowledge, this is the first reported example of the use of this adhesive in flap surgery for pressure ulcers.

**Case presentation:**

We report the case of a 42-year-old Caucasian wheelchair-bound paraplegic man with history of spina bifida, urinary catheter, colostomy, and a history of pressure ulcers. He presented to our institution with a stage IV, methicillin-resistant *Staphylococcus aureus*-contaminated pressure sore on his left ischial tuberosity. A first procedure using V-Y and rotational flap closure dehisced on postoperative day three due to his excessive movement. A second procedure was performed but this also required revision due to dehiscence related to fluid accumulation under the flap. A third procedure using TissuGlu^®^ Surgical Adhesive to adhere the flap and close the dead space resulted in successful resolution. At his last follow-up appointment at seven weeks post-operation he was healing well and was back in his wheelchair.

**Conclusions:**

Any reductions in hospital stay, complication rates, or recurrence rates would be important in this highly problematic group of patients. Elimination of the dead space where fluids can accumulate, combined with adhesion of the flaps with a sufficient strength to withstand the shear forces commonly encountered, could represent an important advancement in the treatment of pressure ulcers requiring surgical repair with myocutaneous or fasciocutaneous flaps. Our initial experience in this case suggests that TissuGlu^®^ may be able to help reduce recurrence rates in this challenging group of patients.

## Introduction

Late-stage pressure ulcers requiring surgical coverage with flaps are common. Various flap techniques have proven effective for defect coverage, but these continue to have high complication and recurrence rates [1–5], leading to additional patient discomfort, scheduling, and management issues for surgical teams and overall high costs of care and treatment [6–8].

Though there is no source for statistics on worldwide procedure volumes, in the United States over 238,000 diagnoses were recorded in 2010 for stage III and IV (Classification of Shea) pressure ulcers according to HCUP (Healthcare Cost and Utilization Project) estimates for ICD-9-CM (International Classification of Diseases, Ninth Revision, Clinical Modification) codes 707.23 and 707.24. The groups most affected are elderly and immobilized patients. Considering the demographic trends and the varying levels of prevention around the world, it is safe to assume that the problem is even more dramatic in many other countries.

Costs and resource usage for these patients are a burden on healthcare systems of any type. In a single-center retrospective study in the Netherlands, Filius *et al*. analyzed ‘direct medical costs of hospital care for surgical treatment of pressure sores stage III and IV’ in a total of 52 cases and identified a range from €20,957 to €40,882, depending on the location of sore [6]. Brem *et al*. reported the average cost for treatment of stage IV pressure ulcers and related complications in 19 patients in New York at $125,000 to $130,000, emphasizing that ‘in calculating costs, the amount spent on treating associated medical complications of pressure ulcers must also be included’ [8].

As these patients require substantially more nursing resources than others, our department is forced to limit the number of these cases admitted, so as not to overburden the staff; handling more than two of these patients at a time becomes logistically challenging.

Complication and recurrence rates are high, driving the costs and resource usage cited above. In a systematic review of the literature on flap surgeries for treatment of pressure sores, Sameem *et al*. reported mean complication rates for myocutaneous flaps at 18.6%, fasciocutaneous flaps at 11.7%, and perforator-based flaps 19.6%, while recurrence rates for the same three groups were 8.9%, 11.2%, and 5.6%, respectively [1]. Other studies have reported complication and recurrence rates of up to 54% and 61%, respectively [2, 9].

The most commonly cited factor in complications and recurrence is wound dehiscence, generally associated with persistent dead space in the wound cavity, shear forces on the tissue planes, and accumulation of serous fluids [1]. Elimination (closure) of the dead space is critical to effective healing, but this is not always easy or possible to obtain with previously described techniques or technologies.

Given the generally poor vascularization and overall tissue quality, suturing techniques to approximate the flaps are usually not an option in pressure sore repair. Filling the dead space with a ‘double adipofascial turnover flap’ has been suggested [10], and may have application in some surgical procedures. It has also been suggested that fibrin sealants may be effective in facilitating wound healing with fasciocutaneous flaps [11]. Erba *et al*. identified a reduced rate of drainage volume in the first postoperative days in the fibrin sealant group [11]. Given that fibrin-based products remain in the wound only for the first one to two days, it is unlikely that these could have an effect on the longer term healing process, where the tissue planes need to be held in approximation for a period of weeks rather than days. Early postoperative drainage volume is unlikely to be a useful indicator of successful wound healing.

A new adhesive technology recently introduced in Germany (TissuGlu^®^ Surgical Adhesive, Cohera Medical, Inc. [Pittsburgh, USA]), is indicated for ‘approximation of tissue layers where subcutaneous dead space exists between the tissue planes in large flap surgical procedures such as abdominoplasty’. Reports on the use of this technology in abdominoplasty suggest that it can eliminate the need for surgical drains in those procedures (Michaels J, unpublished work). Reduced complications and reduced revision rates in inguinal lymph node dissection have also been presented (Stollwerck L, unpublished work). In latissimus dorsi donor site closure at least one group has reported positive experiences in a very challenging patient population, repair of defects related to sternal osteomyelitis (Reutemann M, unpublished work), and several groups have reported on their use of the technology in mastectomy for closure of the flap, in some cases without drain placement [12–14]. Given the mechanism of action described, and the challenges faced in patients with recurrent stage IV pressure sores, we decided to use it to assist with wound closure in a revision flap procedure.

## Case presentation

We report the case of a 42-year-old Caucasian wheelchair-bound paraplegic man with spina bifida who had had surgical treatments over the most recent few years for pressure sores on both the ischial and sacral regions. A supra pubic catheter was placed in 2005 and a colostomy was performed to improve the care after he had suffered from a sacral pressure sore. He was admitted to our clinic because of a stage IV pressure ulcer on his left tuber ischiadicum. The microbiological examination of his wound showed a contamination with methicillin-resistant *Staphylococcus aureus*. His blood test revealed an elevation of inflammatory parameters (14.17×10^3^/μL leucocytes, CRP [C-reactive protein] 10.04mg/dL) and a slightly low protein level at 6.4g/dL. We decided to do a one-stage surgical procedure of debridement and flap coverage using a V-Y procedure and a rotational flap for wound closure. The operation was performed the day after he was admitted to our hospital. We decided not to treat him with antibiotics at this point.

Three days after the first surgical procedure his wound developed a dehiscence due to his excessive movement. A revision, with debridement and surgical closure of the wound, was performed. There were no signs of infection and the results of his blood test showed that the inflammatory parameters were decreasing. The drains showed minor quantities of clear fluid.

**Figure 1:**
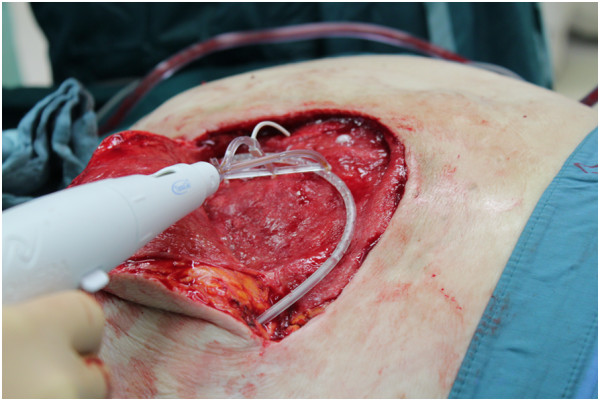
**Pressure sore debrided and rotational flap elevated.** The surgical adhesive is applied to the wound.

**Figure 2:**
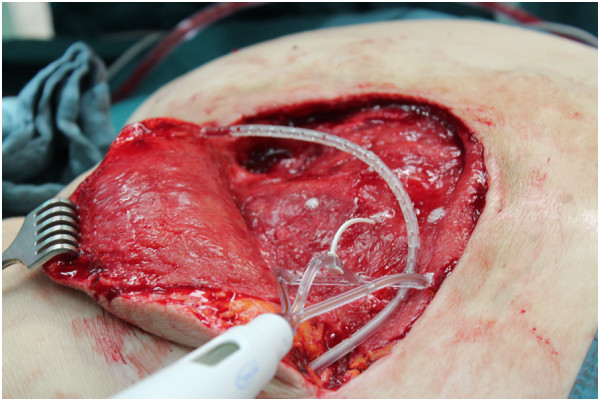
**The end of the application.** The application device allowed the placement of a grit pattern of defined amounts of glue to the wound.

On the eighth day after the second surgery there was a small amount of fluid in his wound dressing, even though the drains were still in place. A new dehiscence of his wound led to a third surgical procedure on day 15 after the first operation. As there was considerable dead space below the flaps we decided, after a debridement and an analysis of his wound situation, to reduce the dead space and hold the flaps in place by using TissuGlu^®^ Surgical Adhesive. This involved the application of a grid pattern of adhesive to the tissue substrate, using a custom applicator device which places measured amounts in a row of three droplets at a time (Figures 1 and 2). The tissue flap was then positioned, attempting to avoid smearing of the grid of drops, and sutured in place as normal. The application of the adhesive took no more than two minutes.

There were no further complications with this pressure sore. He was dismissed 17 days after the last procedure. At seven weeks after the last procedure, he was healing well and was allowed to sit in his wheelchair again.

## Discussion

In a recently concluded retrospective review of 23 patients at our own institution who underwent stage III and IV pressure sore repair with myocutaneous and fasciocutaneous flaps, we found that eight (34.8%) required revision surgery, with three of these requiring two or more procedures. While not inconsistent with the rates reported in the literature, this need for revision surgeries represents an enormous burden on the patients and their families, as well as on the healthcare system and hospital staff.

As part of our efforts to seek techniques or technologies to reduce this recurrence rate, we have analyzed the primary causative factors leading to the need for revision procedures. The underlying, often systemic, wound-healing deficits are exacerbated by two factors: 1) shear forces which cause movement of the flap with respect to the underlying tissue, thereby impeding or interrupting the formation of a collagen matrix; and 2) the accumulation of fluids in the dead space between the tissue planes, which leads to a physical separation of the planes, thereby preventing tissue repair and normal healing. Our search for solutions has therefore been focused on approaches which can effectively hold the two planes in close approximation for the duration of the proliferative phase of wound healing; often significantly longer in pressure sore patients than the 3 to 21 days normally cited for this phase.

Fibrin-based technologies have been extensively studied and have not been shown to be as effective as adhesives for large flap fixation. The facts that the fibrin clots formed do not have a high shear strength and are generally broken down within a few days through fibrinolysis suggest that they may be inappropriate for this particular indication. Techniques of mechanical closure such as quilting sutures have also been studied by many and have generally been found to be effective. Suturing techniques, however, are often not feasible in these wounds due to compromised tissue quality, poor vascularization, and the risk of focal point necrosis. A high-strength tissue adhesive which is biocompatible, non-inflammatory, resorbable, and with a duration of effect of several weeks would correspond to the need.

The adhesive in question is a one-part lysine-based urethane pre-polymer which cures in the presence of moisture. It is applied immediately prior to flap closure using a custom applicator which delivers precisely measured droplets in a grid pattern on the substrate tissue plane. The adhesive drops begin curing on contact with moisture but the process is slow, taking up to 45 to 50 minutes to reach full strength. There is, therefore, no need to rush the process of flap closure. Care should be taken to avoid smearing the drops during closure, and then gentle pressure is applied across the entire flap surface to ensure contact and elimination of dead space. Care must also be taken to avoid pulling the flap as the suture knots are tied, and to avoid any other movement that could interrupt the bonds during the first 40 to 50 minutes after application.

The fully polymerized adhesive droplets maintain their properties of adherence to the tissue for between four and 12 weeks, after which they are slowly broken down through a process of hydrolysis into lysine, CO_2_, and very small amounts of alcohol and polyols (sugar alcohols). Complete resorption can take as long as 24 months. In the event that the revision of a flap is required, drops of adhesive are likely to be identifiable in the wound bed and can be separated from the tissue plane without damage. Re-application of the product in the secondary closure would not be recommended.

## Conclusions

Our case report suggests that this new kind of adhesive may be helpful in attempts to reduce the rates of recurrence and complications in myocutaneous and fasciocutaneous flap surgery for late-stage pressure sores. The application is fast and the costs are acceptable, especially if a recurrence can be prevented. An ongoing prospective series currently being done in our facility will add additional evidence to help evaluate this hypothesis. Further studies will be required before any definitive statements can be made, but the evidence of efficacy of the device in other flap procedures, combined with the logic of closing dead space with points of adhesive, give us reason to be optimistic.

## Consent

Written informed consent was obtained from the patient for publication of this case report and accompanying images. A copy of the written consent is available for review by the Editor-in-Chief of this journal.
